# Supplementary material for: Capture of associated targets on chromatin links long-distance chromatin looping to transcriptional coordination
Source: Nat Commun. 2016 Sep 16;7:12893. doi: 10.1038/ncomms12893 (PMC5028417; doi:10.1038/ncomms12893)
Supplement: Supplementary Information — Supplementary Figures 1-10, Supplementary Tables 1-5 and Supplementary Methods [file ncomms12893-s1.pdf]

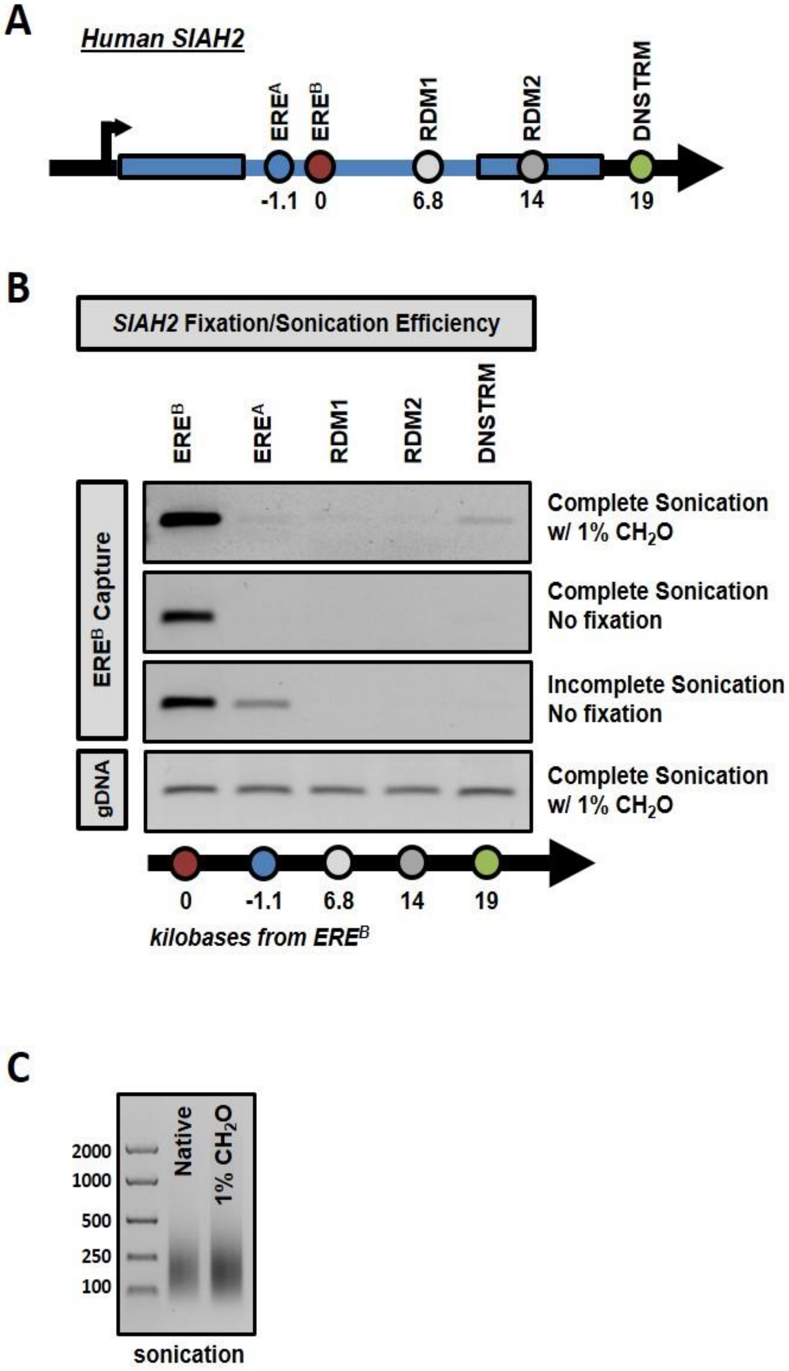

Supplementary Figure 1: (A) This diagram represents the relative location of several key EREs within and around the human *SIAH2* gene that were tested via CATCH in part B. (B) The DNA gel image shows that when specifically capturing the ERE^B locus within the intron of *SIAH2*, incomplete DNA sonication allows capture of a locus approximately 1.1 kb away (ERE^A). In addition, lack of CH_2O fixation results in capture of only the targeted fragment. However, with complete sonication and fixation, the ERE downstream of SIAH2 is also captured with the ERE^B (DNSTRM). (C) This is a DNA gel image showing representative genomic DNA fragment size after complete sonication, using both unfixed (native) and fixed (1% CH_2O) MCF-7 cells.

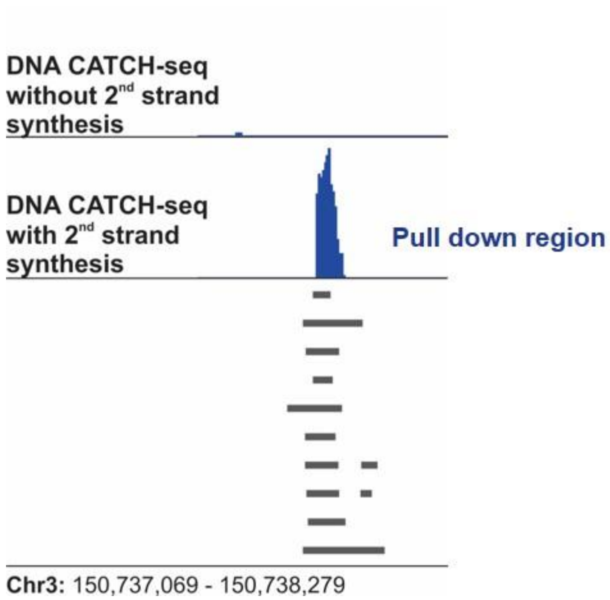

Supplementary Figure 2: These histograms (same scale, Y-axis) show that CATCH-seq requires 2nd strand synthesis to visualize sequencing from the captured region (complimentary to the biotinylated olligo). The presence of the biotinylated oligo during the CATCH protocol prevents that portion of the genome from rehybridizing with its natural complimentary region of DNA. Subsequently, without second strand synthesis, this locus can not be detected by sequencing.

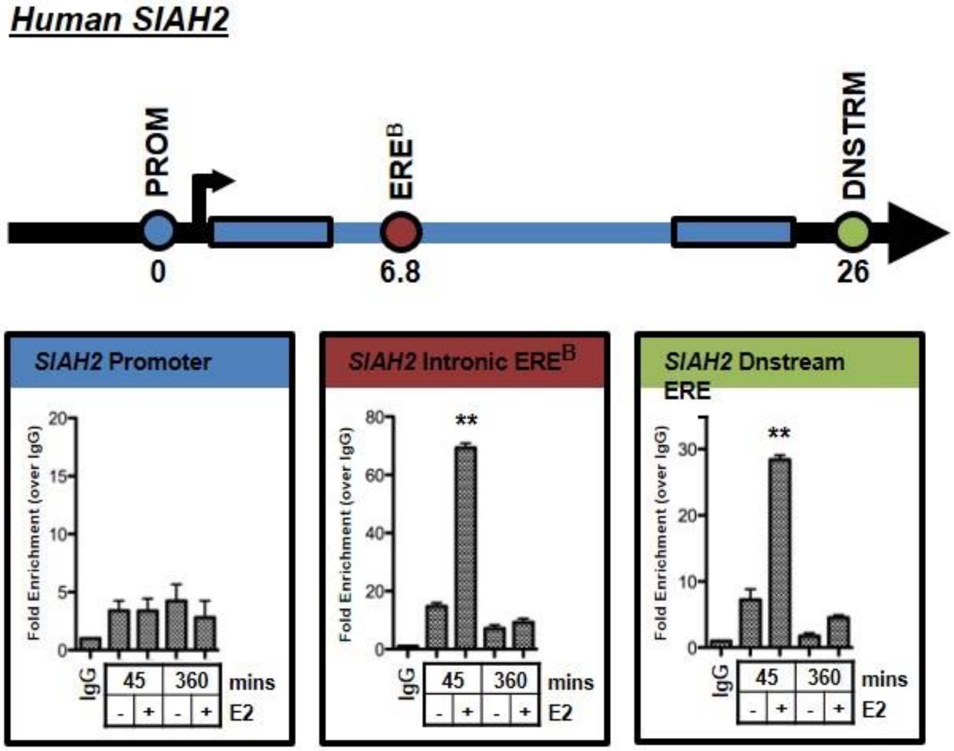

ChIP-qPCR Primers	
**Oligo Name**	**Sequence 5' to 3'**
SIAH2 PROM FWD	GGGTTGTCTTTTCTGGGTTG
SIAH2 PROM REV	GTTGCCTTTCTCGTTTGAGC
SIAH2 EREB FWD	TCCACACATAACTGGCCAAA
SIAH2 EREB REV	CCAATCTTGGGCTGGTATTTTA
SIAH2 DN FWD	CCTAATGGATGGCAACTGCT
SIAH2 DN REV	TTCAGATCAAAGGCCGACTC

Supplementary Figure 3: This is a chromatin immunoprecipitation experiment showing ERα binding at various locations around the *SIAH2* gene before and after E2 treatment. This experiment validated previous work showing ERα binding at the intronic and downstream EREs, as well as showing a lack of ERα binding at the SIAH2 promoter in MCF-7 cells, which corroborates previous findings.

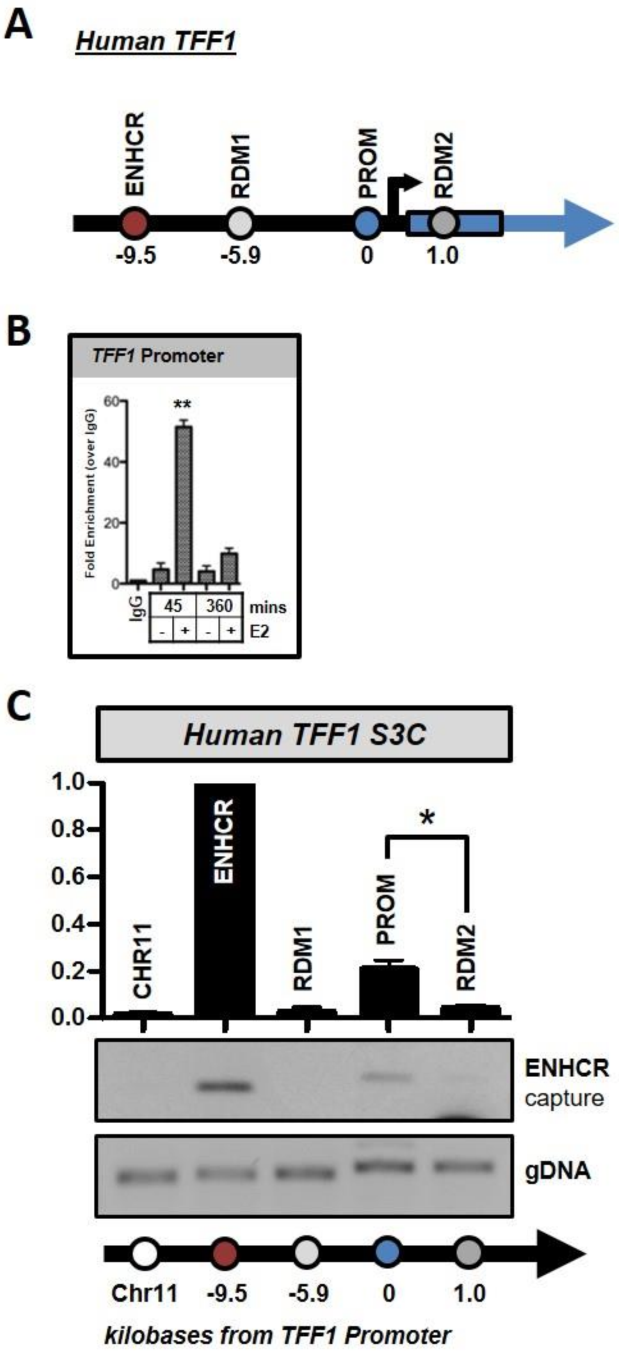

PCR Primers for S3C Product Detection (TFF1)			Oligos for Targeted Enrichment	
**Oligo Name**	**Sequence 5' to 3'**	**PCR Product Size**	**Internal Oligo**	**bp from DNA Target**
CHR11-F<br>CHR11-R	CACACACACAACTATGCCTCA<br>TTCTGACTCTGCCTACCGTT	100	n/a	n/a
TFF1 ENHCR-F<br>TFF1 ENHCR-R	CTTTCGGAGGGCCAGATTCA<br>GACTCATGCTGCTCCCCTC	105	/5BioTinTEG/GGTCCCCCACAAAGTCAGAATTG	184
TFF1 RDM1-F<br>TFF1 RDM1-R	GAACTCAGAGCTCCAAGGGT<br>GGACAAGTAGCCACCTGAGT	108	n/a	n/a
TFF1 PROM-F<br>TFF1 PROM-R	TGGTCAAGCTACATGGAAGGA<br>AAGGTCAGGTTGGAGGAGAC	125	n/a	n/a
TFF1 RDM2-F<br>TFF1 RDM2-R	GGCAGACCGTTGATCCATTC<br>GGTGTTGTCAAGTGGATCGG	125	n/a	n/a

Supplementary Figure 4: **(A)** Human *TFF1* is known to have an ERα-binding enhancer region approximately 9.5 kb upstream of its TSS. This diagram shows the approximate location of that enhancer (red circle), the promoter region (blue circle), and each "random" site (grey circles) tested via CATCH, relative to each other and relative to the *TFF1* gene (blue line). **(B)** ChIP experiment showing the responsiveness of the TFF1 promoter to E2 treatment at 45 minutes. Served to validate previous findings, as well as positive control for E2-responiveness in Supplemental Figure S3. **(C)** The gel image shows the relative intensity of PCR products associated with each tested locus around *TFF1*. The gDNA gel represents the total genomic input to the CATCH protocol, while the ENHCR capture gel represents just the pool of DNA that was enriched after CATCH was done targeting the enhancer region upstream of *TFF1*. The graph displays the mean (SEM error bars) of three experimental replicates. Student's unpaired t-test of data sets with unequal variance was performed to compare the strongest negative control signal against the promoter region (* denotes $p \leq 0.05$).

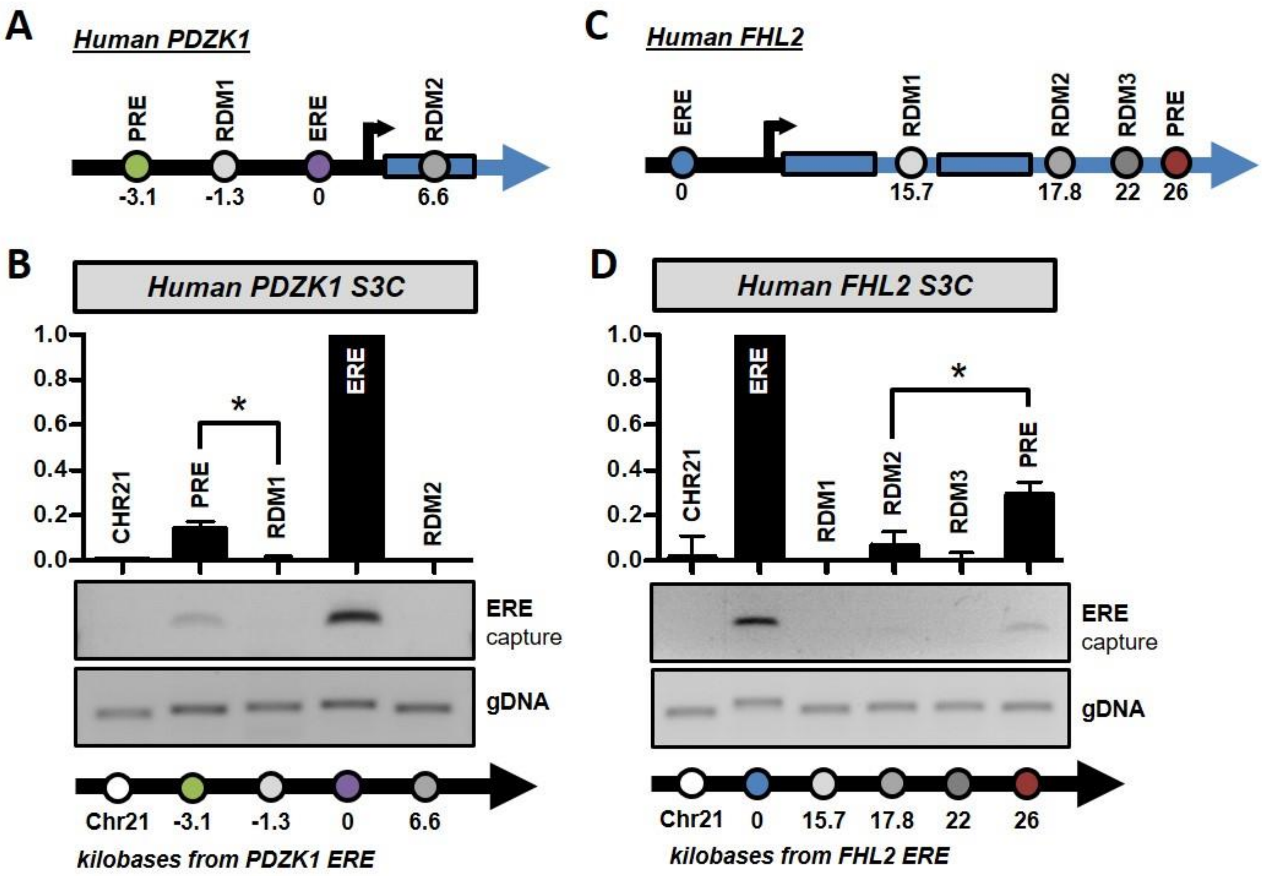

PCR Primers for S3C Product Detection (PDZK1)			Oligos for Targeted Enrichment	
**Oligo Name**	**Sequence 5' to 3'**	**PCR Product Size**	**Internal Oligo**	**bp from DNA Target**
CHR21-F<br>CHR21-R	GGCTTGTTTGAGAGAGCGAG<br>TTAACATTGCCCCTTGTGCC	100	n/a	n/a
PDZK1 PRE-F<br>PDZK1 PRE-R	CAGAGTACACAGTCGCCTCT<br>GCTCCAGTGGTTTTCTCTCC	111	n/a	n/a
PDZK1 RDM1-F<br>PDZK1 RDM1-R	TGGACTCAAGCATTCCTCCC<br>AGCAATCTGGTCAGGAAGCT	118	n/a	n/a
PDZK1 ERE-F<br>PDZK1 ERE-R	GGGATTGCGATGAACTCAGG<br>ACCAGCTTATCTTCCTCCACC	123	/5BioTinTEG/TGAAAGATATAGAGGAGGCCAGGAG	158
PDZK1 RDM2-F<br>PDZK1 RDM2-R	TGCAGGGATTACAGGTGTGA<br>CCCCTGACATTCTAGCTTGGA	114	n/a	n/a

PCR Primers for S3C Product Detection (FHL2)			Oligos for Targeted Enrichment	
**Oligo Name**	**Sequence 5' to 3'**	**PCR Product Size**	**Internal Oligo**	**bp from DNA Target**
CHR21-F<br>CHR21-R	GGCTTGTTTGAGAGAGCGAG<br>TTAACATTGCCCCTTGTGCC	100	n/a	n/a
FHL2 ERE-F<br>FHL2 ERE-R	GTCTAGCCCACCAGCCTC<br>GGGTCTGAGCTGTACAAATGC	129	/5BioTinTEG/CTAGAAGCCCTGCCTTTCTTTGG	175
FHL2 RDM1-F<br>FHL2 RDM1-R	ACACCAGCTATTCCTGTGGT<br>CGCAGTGTGAATAAGCAGCA	110	n/a	n/a
FHL2 RDM2-F<br>FHL2 RDM2-R	CCCTGATCCACCACTGAAGT<br>GTGGGCAGAGATCACATTCG	109	n/a	n/a
FHL2 RDM3-F<br>FHL2 RDM3-R	AAACCCACCCTTCTGTCCTC<br>GCTGGACCCTGAGAATGTGA	105	n/a	n/a
FHL2 PRE-F<br>FHL2 PRE-R	CTGAGAGAAACGTTGCGGAG<br>CAAAAGACAGAGTGCCTTCCA	108	n/a	n/a

Supplementary Figure 5: (A) Human *PDZK1* contains both ER and PR binding sites just upstream of the TSS. This diagram shows the approximate location of both of those sites (PRE: green and ERE: purple), as well as each "random" site (grey circles) tested via CATCH in part B. **(B)** The gel image shows the relative intensity of PCR products associated with each tested locus around *PDZK1*. The gDNA gel represents total genomic input, and the ERE capture visualizes only the pool of DNA that was enriched after CATCH targeting the estrogen binding element just upstream of the *PDZK1* TSS. The graph displays the mean (SEM error bars) of three experimental replicates. Student's unpaired t-test of data sets with unequal variance was performed to compare the strongest negative control signal against that of the PRE (* denotes $p \leq 0.05$). **(C)** The human *FHL2* gene has an ERE located about 5 kb upstream, and a PRE about 21 kb downstream within its second intron. This diagram shows the relative position of the ERE (blue circle), PRE (red circle), and random sites (grey circles) tested in part D. **(D)** The gel image shows the relative intensity of PCR products associated with each tested locus around *FHL2*. The gDNA gel represents total genomic input, and the ERE capture visualizes only the pool of DNA that was enriched after CATCH targeting the estrogen binding element upstream of the *FHL2* TSS. The graph displays the mean (SEM error bars) of three experimental replicates. Student's unpaired t-test of data sets with unequal variance was performed to compare the strongest negative control signal against that of the PRE (* denotes $p \leq 0.05$).

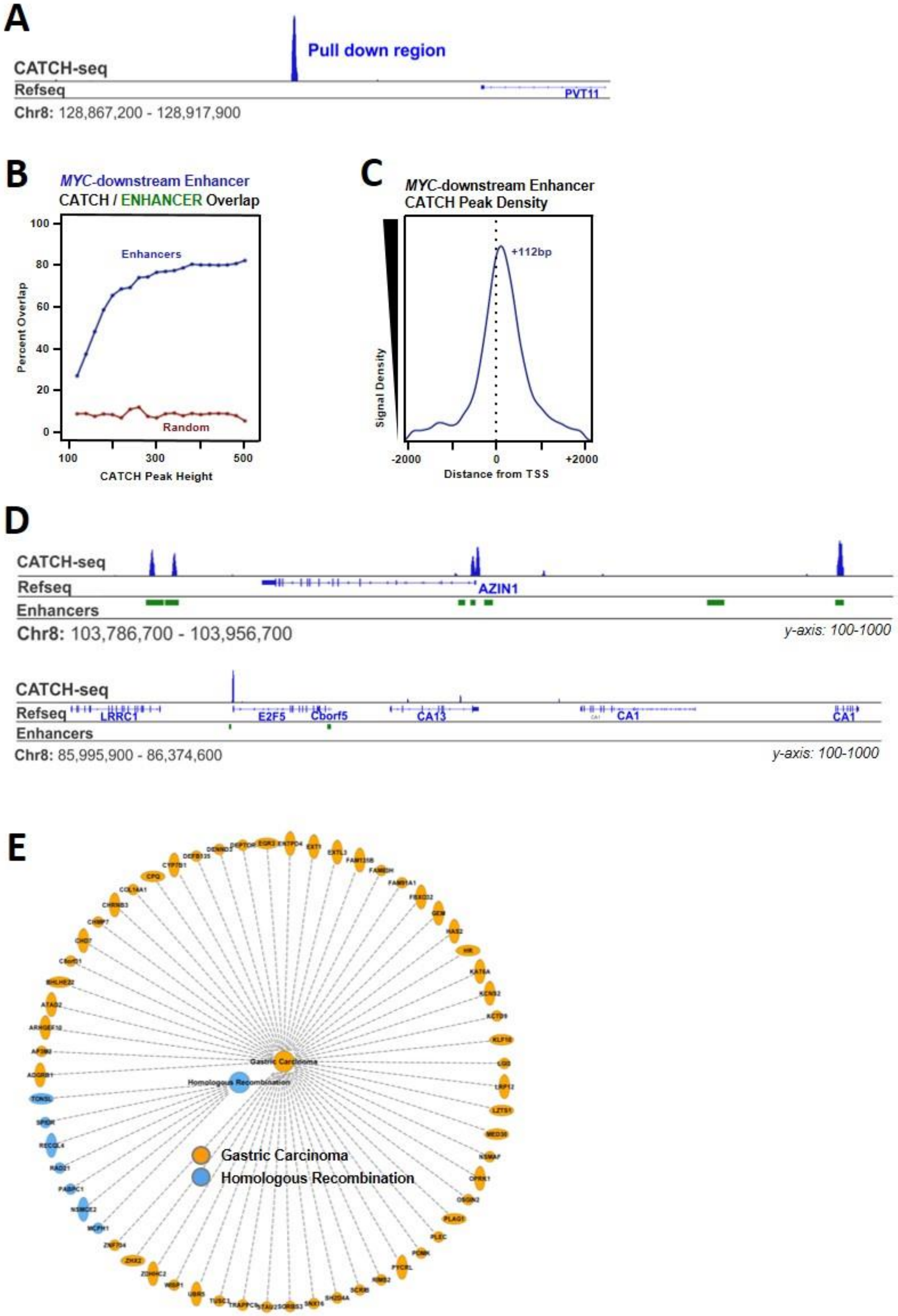
A
CATCH-seq
Pull down region
Refseq
PVT11
Chr8: 128,867,200 - 128,917,900
B
MYC-downstream Enhancer
CATCH / ENHANCER Overlap
Enhancers
Random
Percent Overlap
CATCH Peak Height
C
MYC-downstream Enhancer
CATCH Peak Density
+112bp
Signal Density
Distance from TSS
D
CATCH-seq
Refseq
AZIN1
Enhancers
Chr8: 103,786,700 - 103,956,700
y-axis: 100-1000
CATCH-seq
Refseq
LRRC1
E2F5
C8orf5
CA13
CA1
Enhancers
Chr8: 85,995,900 - 86,374,600
y-axis: 100-1000
E
Gastric Carcinoma
Homologous Recombination

Supplementary Figure 6: (A) An enhancer region downstream of *MYC* was directly captured via CATCH. **(B)** Enhancer downstream of *MYC* CATCH-seq interactions significantly overlap with enhancer marks (blue) compared to statistically randomized control (red); the frequency of these overlaps increase with peak height. **(C)** The CATCH-seq interactions of the enhancer downstream of *MYC* occur, on average, 112 bp downstream of TSS's on chromosome 8. **(D)** Examples of raw CATCH-seq histograms (background subtracted) demonstrating the locations of peaks overlapping with enhancer marks. Additionally, the peak at the E2F5 promoter demonstrates the specificity of CATCH, as no other gene promoters in the region are positive for long-distance interactions. **(E)** IPA analysis detailing specific gene promoters that demonstrated physical interaction with the enhancer downstream of *MYC*. The most significantly enriched process (blue) and disease (orange) enriched within this dataset was homologous recombination and gastric carcinoma, respectively.

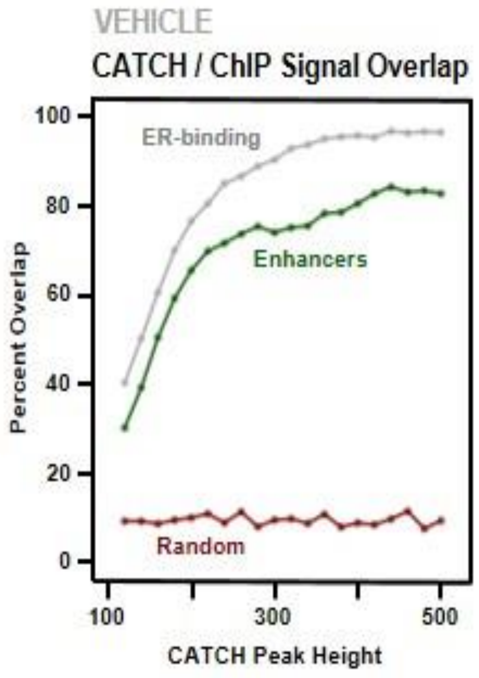

Supplementary Figure 7: *SIAH2* (vehicle-treated) enhancer CATCH-seq interaction peaks significantly overlap with enhancer marks (green) and ER-binding sites (gray) compared to statistically random control (red); the frequency of these overlaps increase with peak height. The same pattern is observed with estradiol treatment in Figure 4E.

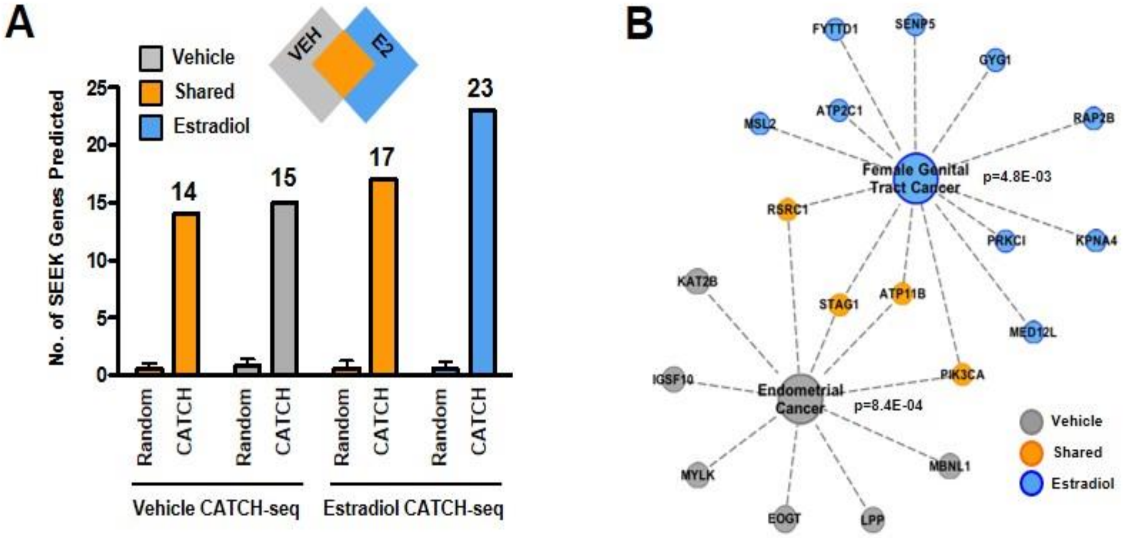

Supplementary Figure 8: (A) Graph detailing the number of SEEK genes 'predicted' by the vehicle-treated (gray) or estradiol-treated versions of *SIAH2* enhancer CATCH-seq genome-wide. Genes predicted by both conditions (the majority) are in orange. **(B)** IPA showing the topmost statistically significant and enriched pathway for each small gene subset identified by CATCH-seq / SEEK overlap: female genital tract cancer (estradiol treated; blue) and endometrial cancer (vehicle treated; gray).

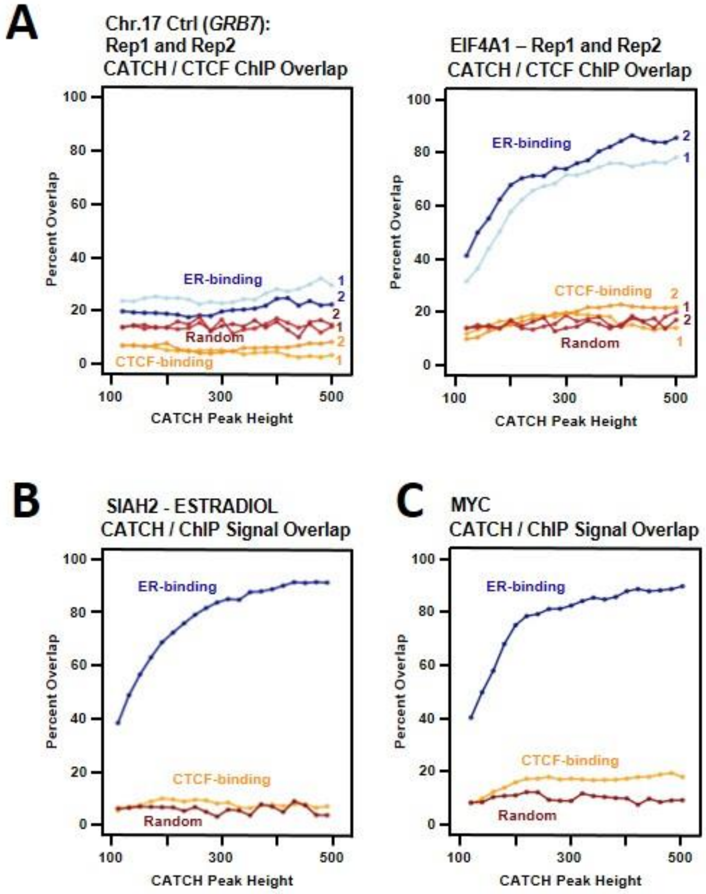

Supplementary Figure 9: Analysis of CTCF binding adjacency/overlap with CATCH peaks. Numbers 1 and 2 denote replicates for *GRB7* and *EIF4A1*. **(A, left)** Control *GRB7* promoter region CATCH-seq interaction peaks do not significantly overlap with CTCF-binding sites (orange) and have very modest overlap with ER-binding sites (blue) compared to statistically random control (red); the frequency of these overlaps does not increase with peak height. **(A, right)** *EIF4A1* promoter region CATCH-seq interaction peaks do not significantly overlap with CTCF-binding sites (orange), but have significant overlap with ER-binding sites (blue) compared to statistically random control (red); the frequency of the latter overlaps increases as a function of peak height. **(B)** *SIAH2* downstream enhancer region CATCH-seq interaction peaks do not significantly overlap with CTCF-binding sites (orange), but have significant overlap with ER-binding sites (blue) compared to statistically random control (red); the frequency of the latter overlaps increases as a function of peak height. **(C)** *MYC* downstream enhancer region CATCH-seq interaction peaks show modest overlap with CTCF-binding sites (orange), but have significant overlap with ER-binding sites (blue) compared to statistically random control (red); the frequency of both overlaps increases, to some degree, as a function of peak height.

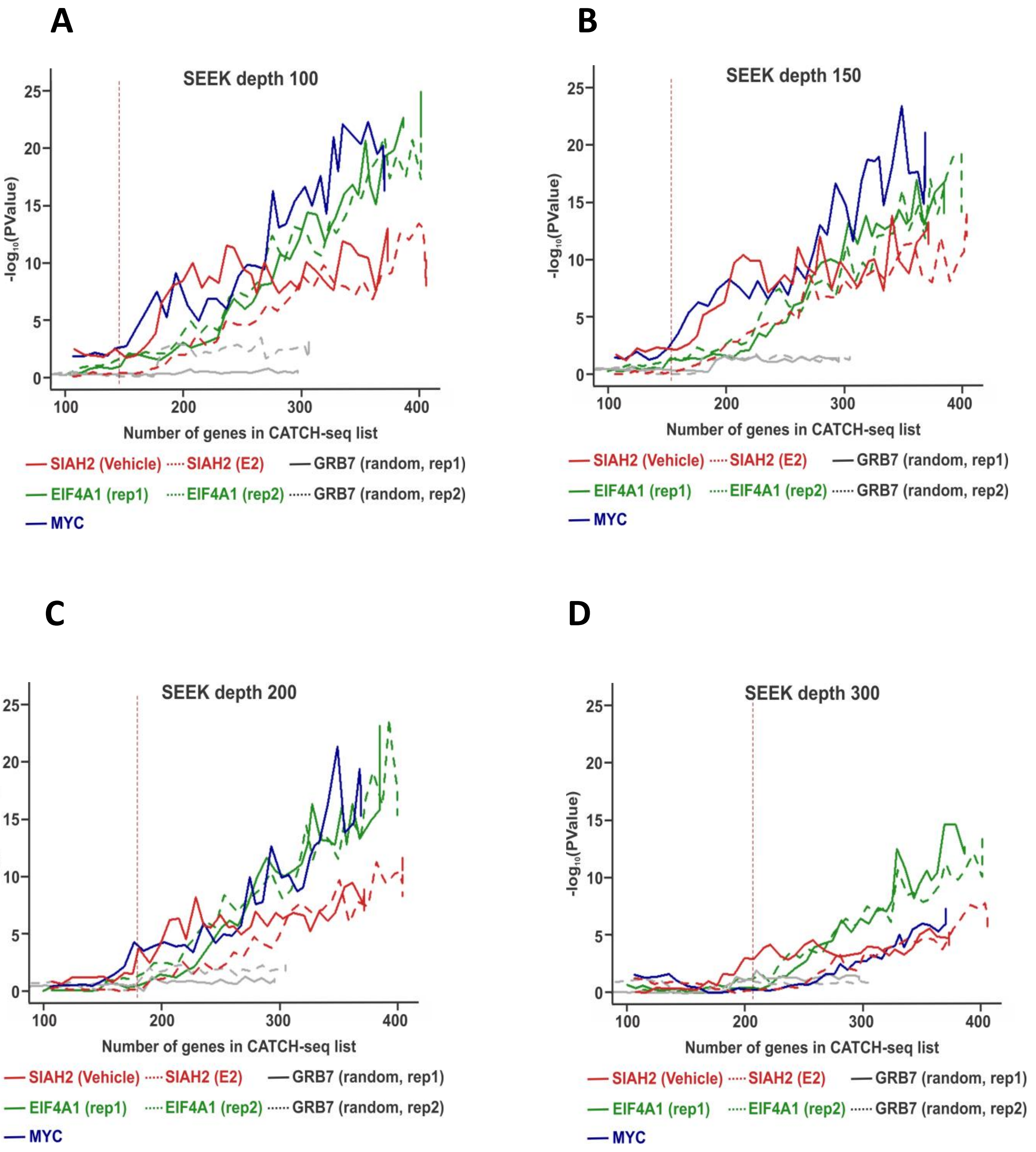
A
SEEK depth 100
-log10(PValue)
Number of genes in CATCH-seq list
B
SEEK depth 150
C
SEEK depth 200
D
SEEK depth 300
SIAH2 (Vehicle)
SIAH2 (E2)
GRB7 (random, rep1)
EIF4A1 (rep1)
EIF4A1 (rep2)
GRB7 (random, rep2)
MYC

Supplementary Figure 10: The graphs depict, using a constant SEEK list of the top 100, 150, 200, or 300 identified genes, the continuous p-value of each CATCH experiment as the number of CATCH genes identified (used) in the analysis decreases. The vertical dotted red lines indicate the average point (of all CATCH experiments on the graph) at which CATCH results become non-significant (p=0.01). As CATCH is used to identify more gene promoters, it's ability to predict transcriptionally co-expressed genes (as measured by SEEK) increases. The negative $\log_{10}$ of the p value was plotted on the y-axis against the number of CATCH genes used on the x-axis. The continuous p value was graphed for every CATCH experiment at varying steady-state SEEK depths of 100 (A), 150 (B), 200 (C) and 300 (D). The p-value here, is the effective point at which CATCH results can no longer, with p degree of certainty, be sure that a given identified gene is reliably co-expressed.

Supplementary Table 1. Basic Comparison of DNA-DNA Interaction Detection Techniques

Technique	Cardinality	Resolution	Advantages	Disadvantages
3C-qPCR	One-to-one	kilobases	Simple	Long protocol, labor intensive, many controls, foreknowledge of interactions required
3C/4C-seq	One-to-all	kilobases	Improved resolution over qPCR, can adapt for higher throughput	Single locus per experiment, more complex data analysis
5C	Many-to-many	kilobases	Can identify interactions between many fragments	Highly labor intensive, not genome-wide, challenging primer design, more complex data analysis
Hi-C	All-to-all	kilo/megabases	Genome-wide, all-to-all interactions	Cost-prohibitive, massive sequencing depth required, poor resolution, advanced bioinformatics required, all-to-all can be confounding
ChIA-PET	Many-to-all	kilobases	Genome-wide detection, enzymatic digestion not required	Requires antibodies, can only detect the interactions resulting from a single protein
T2C	Many-to-all	kilobases	Can detect regional interactions, less expensive than Hi-C or 5C	Restricted to selected genomic regions, advanced bioinformatics required, large primer design effort
**CATCH**	One-to-all	centibases	Most cost effective technique, takes only 24 hours to complete, no enzymatic digestion/ligation, highest resolution/detection	Requires selecting locus to probe (one-to-all), single pulldown locus per experiment

Supplementary Table 2. SEEK list "seed" genes from associated CATCH pulldowns

Chr17 Ctrl *GRB7* 17q12		*EIF4A1* 17p13		*SIAH2* (vehicle) 3q25		*SIAH2* (estradiol) 3q25		*MYC* 8q24.21	
**Gene**	**Cytoband**	**Gene**	**Cytoband**	**Gene**	**Cytoband**	**Gene**	**Cytoband**	**Gene**	**Cytoband**
ZNF385C	17q21.2	*FXR2*	17p13.1	*GYG1*	3q24-25.1	*TSC22D2*	3q25.1	*TRIB1*	8q24.13
KRT31	17q21.2	*SENP3*	17p13	*IGSF10*	3q25.1	*EIF2A*	3q25.1	*FAM84B*	8q24.21
KRT39	17q21.2	*POLR2A*	17p13.1	*MBNL1*	3q25	*MED12L*	3q25.1	*SQLE*	8q24.1

Supplementary Table 3. CATCH pull-down and SEEK gene list details

CATCH pull-down Experiment	chromosomal location of pull-down	Number of total SEEK genes on chromosome	Number of SEEK genes analyzed (top hits; genome)	Number of SEEK genes analyzed from pull-down chr	Number of genes below p value 0.01	Number of SEEK genes predicted by CATCH (below p value 0.01)	Percent SEEK genes predicted
Chr17 Ctrl (*GRB7*)	chr:17q12	993	100	27	26	0	0.0%
EIF4A1	chr:17p13	1001	100	74	67	16	23.9%
MYC	chr:8q24.21	565	100	82	71	46	64.8%
SIAH2 (vehicle)	chr:3q25	939	150	62	49	15	30.6%
SIAH2 (estradiol)	chr:3q25	948	150	70	54	23	42.6%

Supplementary Table 4. Effect of total SEEK genes analyzed on CATCH/SEEK prediction

Sample	No. top SEEK Genes Analyzed	No. SEEK genes on pulldown chr*	Seek genes predicted at random*	SEEK genes predicted by CATCH*	p value
GRB7 (ctrl)	100	26	1.7	0	0.12
GRB7 (ctrl)	200	35	2.5	0	0.1
GRB7 (ctrl)	500	48	4.4	0	0.03
EIF4A1	100	67	9.1	16	3.70E-03
EIF4A1	200	83	11.5	23	8.42E-05
EIF4A1	500	96	17	30	8.72E-04
SIAH2 vehicle	150	49	4.4	15	1.80E-08
SIAH2 vehicle	200	53	5.2	15	4.46E-05
SIAH2 vehicle	500	59	8.5	17	3.00E-03
SIAH2 E2	150	54	8	23	3.60E-13
SIAH2 E2	200	58	9.8	25	4.57E-12
SIAH2 E2	500	71	14.4	34	1.08E-08
MYC	100	71	20	46	2.13E-12
MYC	200	96	29.2	62	2.76E-16
MYC	500	110	37.5	72	4.06E-11

*p value cutoff 0.01

Supplementary Table 5. CATCH-SEEK Gene-expression Prediction					
SIAH2 Enhancer CATCH / Vehicle			SIAH2 Enhancer CATCH / Estradiol		
**Gene**	**Co-exp Value**	**p value**	**Gene**	**Co-exp Value**	**p value**
AADACP1	---	---	*TSC22D2*	---	---
IGSF10	---	---	*EIF2A*	---	---
MBNL1	---	---	*MED12L*	---	---
MFSD1	1.1327	0	*KPNA4*	1.004	0.0001
ARL6IP5	0.9526	0.0026	*STAG1*	0.9164	0.0008
ACAP2	0.9442	0.0041	*CCNL1*	0.8872	0.0014
KAT2B	0.8731	0.0006	*ACAP2*	0.8624	0.0053
PLSCR4	0.8729	0.0059	*FYTTD1*	0.8315	0.0026
PIK3CA	0.8722	0.0081	*PIK3CA*	0.8125	0.0062
STAG1	0.831	0.0082	*DLG1*	0.7784	0.0017
RAB7A	0.7988	0.0062	*TBC1D23*	0.7773	0.0021
ZBTB38	0.7949	0.0013	*ATP11B*	0.7716	0.0013
EOGT	0.793	0.0037	*PRKCI*	0.7609	0.0002
LPP	0.7367	0.0052	*CDV3*	0.7466	0.0015
CDV3	0.7348	0.0054	*ZNF639*	0.7423	0.0005
RSRC1	0.7215	0.0018	*CLDND1*	0.7336	0.0014
ATP11B	0.7129	0.0096	*RSRC1*	0.7299	0.0003
MYLK	0.6905	0.0078	*ATP2C1*	0.7268	0.0071
			RASA2	0.7165	0.0019
			SSR3	0.6475	0.0031
			RAP2B	0.6412	0.0002
			SIAH2	0.6313	0.0006
			LRRC58	0.6276	0.0042
			GYG1	0.6146	0.003
			SENP5	0.6083	0.0049
			MSL2	0.608	0.0067

Supplementary Table 5: Table contains the lists of genes that had both significant CATCH-seq signal peaks within 2kb of their promoters, and were also identified by SEEK as being transcriptionally co-expressed with one another. ***NB:*** *SIAH2* is independently identified (w/ estradiol treatment).

Supplementary Methods

Capture of Associated Targets on Chromatin User Protocol

Part 1: Nuclear isolation, crosslinking and chromatin shearing

1. Be sure that cells are in log-phase growth.
2. Aspirate cell-growth media.
3. Add 2% Formaldehyde (in serum-free RPMI) (2 ml 16% formaldehyde per 14 ml PBS) to cell culture.
4. Incubate for 10 min at 25°C, gently rocking.
5. Aspirate 2% formaldehyde solution.
6. Quench crosslinking by adding 10 ml of 250 mM Tris-HCl pH 8.0 (in PBS).
7. Incubate for 5 min at 25°C, gently rocking.
8. Scrape cells in 1.0 ml PBS to harvest.
9. Centrifuge cells at 1,500 x g for 6 minutes and aspirate supernatant.
10. Wash cell pellet in PBS and resuspend in 500 µl Sucrose Buffer + PIC.
11. Dounce homogenize 30 times with a tight fitting pestle.
12. Centrifuge for 10 minutes at 6500 RPM at 4°C.
13. Aspirate supernatant and resuspend pellet again in 120 µl of CATCH buffer + PIC.
14. Sonicate samples (BioRuptor, 2 x 7 minutes on High, 30s on/off).
15. Pellet debris at 14,000 RPM for 10 minutes at 4°C.
16. Transfer supernatant to a new 1.5 ml tube.

Part 2: Pre-clearing and hybridization

1. Incubate the chromatin sample at 58°C for 5 minutes to unmask endogenous biotin.
2. Add 10 µl of pre-equilibrated (in CATCH buffer) streptavidin magnetic beads to the sample.
3. Incubate 60 min at 25°C while rotating.
4. Pull out magnetic beads and transfer cleared chromatin supernatant to a PCR tube.
5. Add biotinylated oligo probe (300 nM final conc.) to the chromatin sample.
6. Hybridize the probe to the chromatin as follows *(**blue** step should be at least 5°C above the T_m of the oligo; **red** step should be done below the T_m of the oligo):*

    25°C for 1 min *(equilibration)*
    81°C for 4 min *(gDNA denaturation)*
    72°C - **50°C** decreasing gradient *(15 sec per 1°C)*
    42°C for 10 min *(hybridization)*
    25°C hold

7. Remove unhybridized oligo and other impurities with illustra sephacryl (S-400 HR) microspin column according to manufacturer's instructions.
8. Transfer sample to a new 1.5 ml tube and add 300 µl of DNase/RNase free H_2O.
9. Add 25 µl of pre-equilibrated (in CATCH buffer) streptavidin magnetic beads to the sample.
10. Incubate for 60 min at 25°C while rotating.

Part 3: De-crosslinking and protein digestion

1. Immobilize beads on magnetic stand, aspirate unhybridized supernatant and wash samples 3x in CATCH buffer at 42°C while shaking at 1000 rpm on a thermomixer.
2. Resuspend beads in 150 µl of De-crosslinking buffer supplemented with 1 µl of 20 mg/ml RNase A.
3. Incubate at 55°C while shaking at 700 rpm on a thermomixer for 60 minutes.
4. Add 5 µl of 20 mg/ml Proteinase K to each sample.

USER PROTOCOL

5. Incubate at 65°C while shaking at 700 rpm on a thermomixer for 3-6 hr (or overnight) to de-crosslink.

6. Incubate at 100°C for 1 minute.
7. Remove magnetic beads and transfer DNA sample to a new 1.5 ml tube.

Part 4: DNA Purification

1. Extract once with Phenol:Chloroform:IAA.
2. Extract again with Phenol:Chloroform:IAA, and then once with Chloroform to remove any residual Phenol.
3. Add 10 µg glycogen, 1/10 volume of 3M Sodium Acetate pH 5.2, and 2.5 volumes of 200 proof ethanol.
4. Precipitate DNA at -20°C or at -80°C for 2 hours.
5. Centrifuge at 14,000 rpm for 30 minutes, wash with 70% ethanol and dry pellet.
6. Resuspend the DNA pellet in 30 µl of 10mM Tris-Cl pH 8.5 or nuclease-free H_2O.
7. Proceed immediately to 2nd strand sythesis.

CATCH Buffer (40 ml)

- 400 µl of 1.0 M HEPES (10 mM)
- 240 µl of 5.0 M NaCl (30 mM)
- 160 µl of 0.5 M EDTA pH 8.0 (2 mM)
- 400 µl of 20% SDS (0.2%)
- H_2O to 40 ml
- Add Protease Inhibitors fresh

De-crosslinking Buffer (10 ml)

- 300 µl of 1.0 M Tris-Cl pH 8.0 (30 mM)
- 60 µl of 5.0 M NaCl (30 mM)
- 10 µl of 0.5 M EDTA (0.5 mM)
- 2 µl of of 0.5 M EGTA (0.1 mM)
- 500 µl of 20% (w/v) SDS (1%)
- H_2O to 10 ml
- Add RNase and Proteinase K (fresh)

Sucrose Buffer (10 ml)

- 100 µl of 1.0 M HEPES pH 8.0 (10 mM)
- 1.03 g of Sucrose (0.3 M)
- 30 µl of 1.0 M $CaCl_2$ (3 mM)
- 20 µl of of 1.0 M MgOAc (2 mM)
- 100 µl of Triton-X100 (1%)
- H_2O to 10 ml
